# Biofouled Micro- and Nanoplastics as Reactive Platforms for Potentially Toxic Element Transformation

**DOI:** 10.1021/acs.est.5c15116

**Published:** 2026-04-09

**Authors:** Swaroop Chakraborty

**Affiliations:** School of Geography, Earth & Environmental Sciences, 1724 University of Birmingham, Edgbaston B15 2TT, U.K.

**Keywords:** microplastics, nanoplastics, biofouling, plastisphere, eco-corona, metal speciation, redox transformations

## Abstract

Micro- and nanoplastics (MNPs) are increasingly recognized not only as physical pollutants but also as dynamic chemical platforms. Upon environmental release, they are rapidly colonized by microbial biofilms (the plastisphere) biomolecules and natural organic matter (eco-corona) in the environment, transforming them from inert particles into mobile microreactors. These biofoulings provide redox-active constituents, photoreactive pigments, and ligand-rich polymers that can drive contaminant transformations (e.g., chromium redox cycling, arsenic oxidation, and mercury methylation). Such processes can alter contaminant speciation, mobility, and toxicity, yet they remain absent from most risk frameworks. For example, monitoring and risk assessments typically quantify MNPs by particle counts/mass and polymer identity (and occasionally total metal loads) but rarely measure speciation (e.g., MeHg vs Hg­(II) or As­(III)/As­(V)) on biofouled MNPs. This perspective argues for recognizing MNPs as active pollutant platforms and outlines priorities to quantify biofouled MNP reactivity relative to natural particles, identify and test the toxicity of novel biofouled–metal–plastic complexes, and embed speciation-focused monitoring in policy. By integrating chemistry, ecology, health, climate, and environmental justice, this perspective discusses a forward-looking research and governance agenda to address this overlooked dimension of plastic pollution in a changing world.

## Graphical abstract

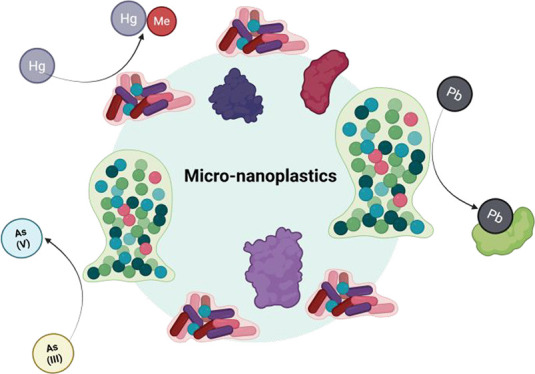

## Introduction

Micro- and nanoplastics (MNPs) have long been viewed as passive “toxin taxis”—inert vectors transporting pollutants like heavy metals and metalloids through the environment.^1^ Indeed, field surveys report that plastic debris is often contaminated with cadmium, lead, arsenic, and other potentially toxic elements (PTEs) in oceans, lakes, and soils.^2^ However, this established narrative overlooks a critical twist: once biofouled, MNPs become dynamic chemical reactors rather than inert carriers. In natural waters, MNPs are rarely the pristine, spherical pellets used in lab experiments.^3^ Within hours of release, they acquire a “plastisphere biofilm”— a complex biofilm of bacteria, algae, sticky extracellular polymers, and eco-corona (composed of natural organic matters, biomolecules, etc.). This biomolecular coating is not merely passive: it imbues plastics with new chemistry (hereby termed as biofouling).

Biofoulings are not unique to plastics: in natural waters and sediments, they form on minerals, detritus, and other solids, where EPS-rich matrices create chemically reactive microenvironments that bind metals and sustain redox/pH gradients.^4,5^ Plastics differ, however, because they are persistent, mobile particles with polymer-specific surface chemistry and additives, they can concentrate hydrophobic chemicals via partitioning into the polymer phase, and they undergo weathering that alters surface functional groups and photochemical reactivity.^6–8^ Together, these features make biofouled MNPs unusually effective as transportable, reactive interfaces compared with many natural particulates. These coatings imbue the particle with new reactive properties. It provides redox-active minerals and metabolites, light-harvesting pigments, and a soup of ligand-rich biopolymers (extracellular polymeric substances, EPS) at the plastic interface. In other words, a once-inert microplastic is transformed into a mobile microreactor where adsorbed contaminants can undergo chemical changes. Recent studies indicate that the presence of MNPs can alter the speciation, mobility, and toxicity of metal^9^—an aspect not captured by traditional risk assessments. Therefore, rather than merely shuttling pollutants unchanged, biofouled MNPs actively influence contaminant chemistry through biofilm-driven redox reactions, photochemical transformations, and strong biochelation processes.

In environmental systems, biofouled MNPs should be considered as one component of a broader particulate continuum that also includes suspended sediments, mineral particles, detrital organic matter, and natural biofilm-coated surfaces. I do not assume that MNPs are the dominant reactive substrate in all settings. Their relevance is likely to be context-dependent: they may be minor contributors where natural particulates dominate particle mass and interfacial area but disproportionately important where small plastic particles remain mobile across compartments, accumulate biofilms, and intersect with efficient biological exposure routes. This distinction is supported by recent comparative work. For example, river biofilms can retain substantially more microplastics than adjacent water and increase particle capture and protist ingestion, supporting a plausible pathway for localized exposure enhancement. By contrast, comparative studies also show that natural particles can match or exceed plastics in contaminant-vector or sorption roles under realistic conditions (e.g., biofilm-covered sand and microplastics showing similarPolychlorinated biphenyls (PCB) transfer to sea urchins and river sediment outperforming polystyrene (PS)/ low-density polyethylene (LDPE) for arsenic adsorption). The perspective, therefore, treats biofouled MNP as conditional, not universal, hotspots of potentially toxic element reactivity and transfer.^10–13^

Countless studies confirm that plastic debris readily adsorbs heavy metals and metalloids.^14,15^ For example, MNPs have been described as “carriers for heavy metals” with surface charge, hydrophobicity, and aging^3^ controlling adsorption. Experiments show that plastics can bind Cd, Pb, Cr,^16^ and metalloids like As or Sb via electrostatic and coprecipitation mechanisms, raising justifiable concern about plastics shuttling these contaminants into food webs.^17^ Environmental surveys have indeed detected toxic metals on aged plastics in freshwater and marine sediments.^18^ However, adsorption is only the first phase; once particles are biofouled, interfacial processes can alter the speciation and fate of associated metals.^19,20^ These biofoulings turn each particle into a tiny chemical reactor, capable of altering the speciation and fate of adsorbed metals. The key distinction from natural particles is the plastic core’s persistence and mobility, which allows these reactor functions to be sustained and transported over long distances and time scales.

Beyond PTEs, MNPs also co-occur with and sorb organic pollutants and contaminants of emerging concern (e.g., pharmaceuticals, personal-care products, pesticides, and per- and polyfluoroalkyl substances­(PFAS)). Adsorption can proceed via hydrophobic partitioning, electrostatic interactions, hydrogen bonding, and π–π interactions, and is strongly modulated by polymer type, aging, and water chemistry.^21–23^ Importantly, biofouling can substantially enhance sorption of organics (including PFAS) under environmentally relevant conditions, extending the “mobile microreactor” concept beyond metals.^24,25^ Because a comprehensive synthesis of MNP-associated transformation of organic pollutants (e.g., PFAS, antibiotics, and dyes), including photochemical mechanisms and transformation products, would go beyond the scope of this perspective, the focus here will be on biofouled MNP-driven transformations of PTEs.

## Biofouling Creates a Dynamic Reactor Interface

Within a biofouled-MNP particle, the following features converge: (1) **Redox-active constituents:** Biofilms often contain iron- or manganese-oxide minerals and redox-active organics (quinones, humic-like compounds, and organic radicals) that can shuttle electrons.^26,27^ These can drive metal redox reactions at the particle surface. (2) **Photochemistry from embedded organisms:** Many plastisphere microbes are photosynthetic or produce photoreactive pigments. Under sunlight or ambient light, biofilms can generate reactive oxygen species (ROS) or excited molecules.^8^ Such in situ photochemistry can oxidize bound metals or change their chemistry. (3) **Ligand-rich microenvironment**. EPS in biofilms are brimming with functional groups - carboxyl, hydroxyl, amino, phosphates and even thiols – that bind metals strongly. Indeed, one study lists EPS moieties (−COOH, −CONH_2_, −SH, −PO_4,_ etc.), which are all excellent metal ligands.^28^ These negatively charged groups and sulfur donors can complex cations (Cd, Pb, Cu, etc.) tightly, altering solubility and bioavailability. Anionic surface functionalities (e.g., deprotonated carboxylates, −COO^–^) can enhance uptake of cationic metals via electrostatic attraction and ion exchange, whereas polar but typically nonanionic groups such as amides (−CONH_2_) contribute through coordination (primarily via the carbonyl oxygen), hydrogen bonding, and dipole–ion interactions. Together, these features create a chemically active interfacial microenvironment on the biofouled particles.

### Environmental Identity Is Interfacial and Method-Sensitive

The “environmental identity” of an MNP is defined not only by polymer type and size but by the acquired **eco-corona**, plastisphere community function, and associated metal pools, which together control binding motifs, redox microenvironments, and bioaccessibility. This identity is therefore **method-sensitive**: if isolation removes the eco-corona or collapses redox gradients, the measured particle is no longer the environmentally experienced entity. I, therefore, treat eco-corona/plastisphere preservation and characterization as integral to any framework that links MNP-associated chemistry to exposure or policy.

## Mechanisms of Biofouling-Driven Overlooked Transformations on MNPs

Several mechanisms are well supported in the broader biofilm and biogeochemical literature, but remain less directly quantified on biofouled MNPs. I, therefore, distinguish MNP-specific evidence from testable hypotheses and use four pathways as illustrative examples. I distinguish throughout this section between (i) **established MNP-specific evidence** (processes directly observed on plastics or plastic-associated interfaces), (ii) **indirect evidence** (mechanisms established in natural biofilms, sediments, or mineral-associated systems and applied here by analogy), and (iii) **testable hypotheses** (mechanistically plausible pathways that remain to be directly validated on biofouled MNPs under environmentally realistic conditions). This distinction is used to clarify where the current evidence base is strong and where the discussion is intentionally forward-looking. Several illustrative pathways highlight the potential:

### Chromium (Cr): Cr­(VI) to Cr­(III)–Evidence Basis

Predominantly **indirect evidence** from natural biofilms/sediments; MNP-specific validation remains limited. Reduction of Cr­(VI) to Cr­(III) generally reduces mobility and acute toxicity; certain Cr (III) complexes can interact with biomolecules, but the overall genotoxic risk is typically lower than for Cr­(VI).^29^ Laboratory studies on biofilm-coated surfaces show rapid Cr­(VI) reduction under mild conditions, suggesting that similar behavior could occur on plastics.^30^

### Arsenic (As): As (III) to As (V)–Evidence Basis

**Mixed evidence** includes direct evidence for redox-active plastic surfaces in some systems and indirect evidence for biofilm-mediated pathways. As­(III) → As­(V) transformation on MNP-associated interfaces is plausible but not yet broadly verified on plastisphere; however, weathered MNPs can mediate arsenite oxidation via redox-active surface moieties that generate H_2_O_2_/ROS, providing a direct MNP-linked mechanism.^31^ The more toxic arsenate (As­(V)) could be produced when As (III) (often bound to plastic) encounters ROS generated by photosynthetic biofilms. For example, UV-activated organic molecules and algal photosystems create hydrogen peroxide or singlet oxygen that oxidize As­(III) to As­(V).^32,33^ As­(V) may be more harmful to some primary producers due to uptake via phosphate pathways, whereas relative toxicity is species- and context-dependent. Relative sensitivity to As­(III) versus As­(V) varies with physiology, uptake pathways, and detoxification capacity, so speciation changes may shift risk differently across taxa and life stages.

### Lead (Pb): Particulate Pb to Soluble Pb–Organic Complexes–Evidence Basis

Primarily indirect evidence from EPS-metal complexation and biofilm chemistry, with specific MNP-interface effects requiring direct speciation-focused testing. Lead adsorbed as insoluble salts or oxides on a plastic surface may be solubilized by EPS ligands. Carboxyl-rich EPS (from bacteria or algal coatings) can chelate Pb^2+^ to form Pb-citrate or Pb-oxalate complexes. In one study, EPS–Pb complexes can increase bioavailability relative to inorganic Pb solids.^28^ Pb solubilization on MNP plastisphere is hypothesized and likely system-dependent; current evidence more robustly supports MNP-associated shifts in Pb partitioning/speciation (e.g., changes in organic-bound Pb fractions) under soil incubation conditions, which can indirectly alter mobility and bioavailability.^34^

### Mercury (Hg): Hg­(II) to MeHg (Methylmercury)–Evidence Basis

Predominantly **indirect evidence** (biofilm/periphyton methylation literature), with MNP-linked pathways treated here as **testable hypotheses** unless directly demonstrated. Mercury methylation is established in aquatic biofilms/periphyton, where anoxic microniches and the biofilm structure can support net methylmercury production and measurable methylation/demethylation dynamics. Whether biofouled MNPs generates comparable net methylation rates under environmentally realistic particle loadings remains insufficiently quantified and should be treated as a testable hypothesis requiring MNP-specific tracer-rate measurements and comparison to sediment/periphyton baselines.^35^ Biofilms frequently harbor sulfate-reducing and other anaerobic bacteria capable of methylating inorganic mercury. MNP surfaces laden with biofilm sulfides create anoxic microzones ideal for methylators. Indeed, biofilm cultures often show substantially higher methylation rates than planktonic cultures.^36^ This raises the important possibility that plastics collecting Hg (II) in sediments could become hotspots for producing neurotoxic methylmercury, which then enters food chains. Additional paddy-soil experiments report MNPs influencing bioavailable Hg­(II)/MeHg dynamics, supporting an MNP-relevant effect while highlighting that net rates and field-scale contributions remain to be quantified.^37^

Each case illustrates how a seemingly “low-risk” contaminant could be transformed into a more hazardous form when associated with biofouled surfaces. For example, otherwise “low-risk” As­(III) adsorbed onto MNPs may, upon exposure to redox-active plastic surfaces—especially during oxic–anoxic fluctuations—be oxidized to more toxic As­(V), as recent studies show that oxidized functional groups on MNPs can mediate arsenite oxidation.^31^ Similarly, Pb immobilized in sediments could be remobilized via ligand exchange with biofilm-derived EPS, which significantly alters adsorption–desorption behavior at the MNP interface. Such transformations, though speculative, are chemically plausible and warrant consideration in risk assessments.^38^ Importantly, speciation change does not necessarily increase toxicity; the exposure consequence depends on whether transformed species remain MNP-associated (e.g., sorbed or EPS-complexed) or are released to water/porewater, thereby altering bioavailability and uptake routes, a system-dependent uncertainty that warrants direct measurement. Such speculative but plausible pathways suggest that biofouled MNPs may act as catalysts of contaminant cycling in ways that current monitoring frameworks and safety sheets do not anticipate. While MNPs can provide transportable colonization surfaces and concentrate reactants, the system-level significance of MNP-driven PTE transformations cannot yet be generalized because (i) few studies report rate constants (not only sorption), (ii) results are rarely normalized to surface area and biofilm biomass, and (iii) there are limited mass-balance comparisons against dominant natural particle surfaces (sediments/clays/periphyton). Priority next steps include isotope-tracer quantification of transformation rates on the MNP-associated plastisphere, microprofile mapping of redox/pH within the plastisphere, and side-by-side baselines with natural particulates under matched conditions.

## Why This Matters: Cascading Environmental Threats

These transformations may affect exposure and hazards in several ways.

### Unpredictable Toxicity

Transformations can switch a pollutant’s hazard level. For instance, relatively less toxic Cr­(III) on biofouled MNPs could reoxidize to Cr­(VI) in a photoactive biofilm or mild As (III) can convert to more toxic As­(V) under sunlight and ROS. As a result, particles may transport contaminants whose speciation has changed during environmental transit, with possible consequences for toxicity and bioavailability.

### Localized Reactive Microenvironments

Clusters of biofouled MNPs may create localized zones of enhanced interfacial reactivity. Microenvironments inside biofilms can have very high [H^+^] (low pH) or reactive oxygen concentrations relative to that of bulk water. In such niches, reaction rates increase substantially. In these microenvironments, interfacial redox and acid-driven processes may proceed differently from those inferred from bulk water chemistry alone.

### Bioavailability Spikes and Pollution Mobilization

EPS–metal complexes may enhance cellular uptake via ligand-assisted transport or endocytosis, although mechanisms remain to be confirmed. If biofouled MNP surfaces convert metals into soluble organo-complexes, they may alter exposure pathways relative to simple ionic metal forms. Plastics may mobilize contaminants previously stabilized in sediments, thereby extending the environmental persistence of legacy pollutants. Whether these processes increase risk in practice depends on speciation, release to water/porewater, and biological uptake routes. Importantly, none of these threats is captured by standard measurements of MNP pollution. If monitoring only reports total metal loads on plastics, it misses what fraction is transformed into new species. Future scenarios highlight how these transformations may intensify under global change. As Arctic Sea ice retreats, plastisphere- or eco-corona-acquired particles could become new hotspots for mercury methylation^39,40^ in polar food webs. Similarly, tropical cyclones and storm surges may resuspend sediment-buried plastics and potentially remobilize legacy arsenic or lead pools. Nanoplastics, owing to their size and mobility, may penetrate new biological compartments, potentially facilitating transformations directly within tissues. These scenarios are plausible but remain hypotheses that require direct validation.

## The Critical Knowledge Gaps

Despite the growing focus on MNP pollution, our understanding of their role as biofilm-mediated chemical reactors remains limited by several fundamental blind spots. Most studies still report only the total contaminant load associated with plastics, without assessing speciation, oxidation state, or the identity of transformation products, factors that ultimately govern toxicity and mobility. Laboratory experiments further compound this gap by relying predominantly on pristine, spherical beads, which poorly represent the heterogeneous, weathered, and biofilm-colonized plastics found in nature. While biofilm-driven processes such as mercury methylation or chromium reduction are well established on natural particles, direct validation of these mechanisms on biofouled MNPs is very limited, leaving open the question of whether plastics uniquely amplify or modulate such reactions. Equally concerning is the absence of detailed characterization of novel products that may arise at the MNP–biofilm interface, such as EPS–metal–plastic complexes, for which neither structural identities nor toxicological profiles are currently known. Box 1 in Figure 1 distils these into the most urgent, actionable priorities for the next 3–5 years, including methodological innovations needed to quantify MNP-associated transformation rates.

**Figure 1:**
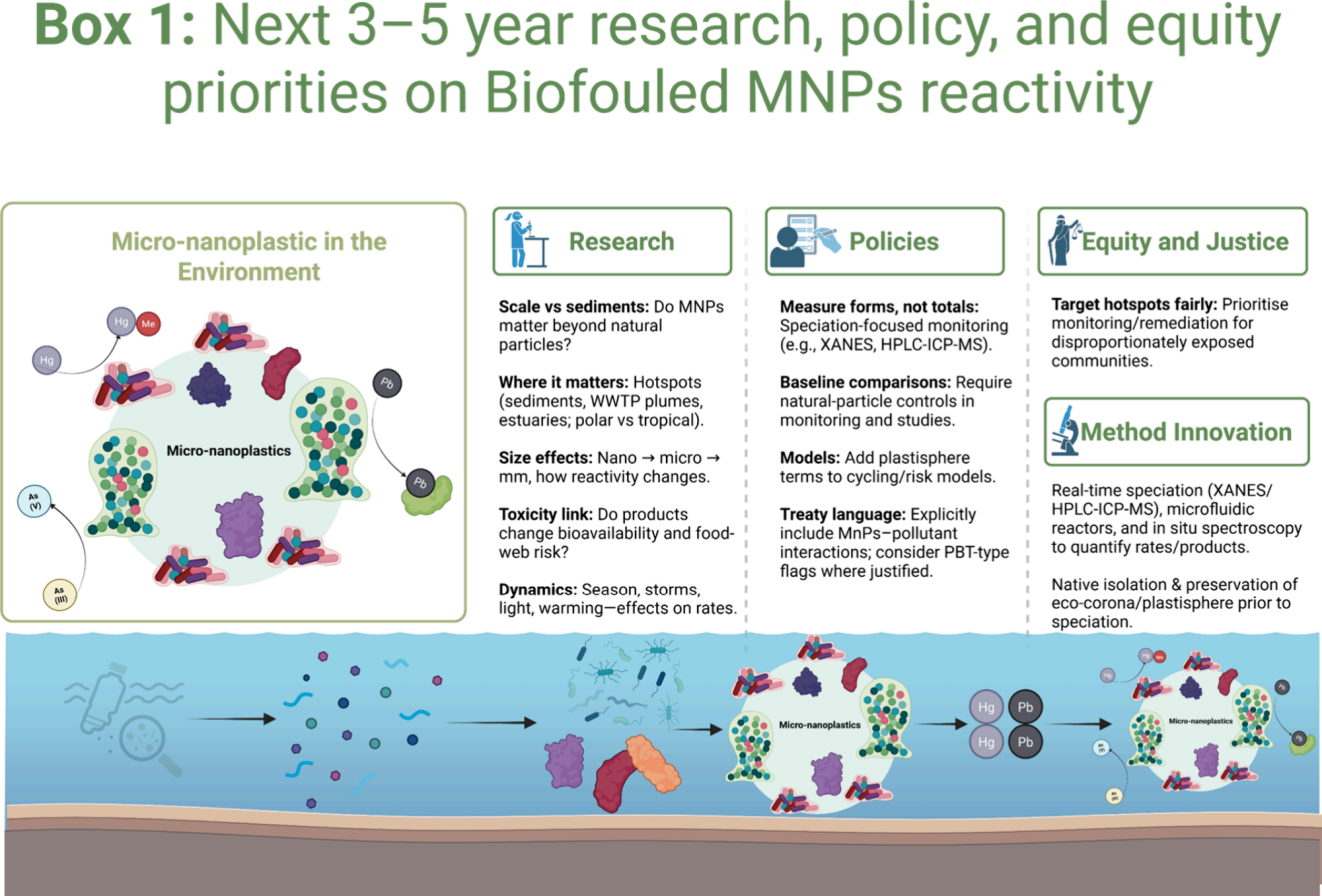
Box 1 | Research, policy, and equity priorities on biofouled MNP reactivity. Conceptual overview showing how micro- and nanoplastics in aquatic systems acquire an eco-corona/plastisphere biofilm that concentrates PTEs and can alter their chemical form (e.g., Hg methylation, As redox transformations, and Pb complexation with EPS). The lower schematic illustrates progression from plastic inputs to dispersed particles, biofouling/aggregation, and colocalization with PTEs before downstream exposure and risk. The three panels (“Research”, “Policies”, and “Equity and Justice, Method Innovation”) summarize key knowledge gaps, actionable monitoring/model priorities, and the need to prioritize disproportionately exposed communities in assessment and remediation. Created in BioRender. Chakraborty, S. (2026) https://BioRender.com/objfsmx.

A central unresolved question is the comparative significance of biofouled MNP-driven transformations relative to natural particulates, such as clays, sediments, and organic detritus. While these substrates are far more abundant and reactive, plastics’ persistence, buoyancy, and global ubiquity may allow them to act as disproportionately mobile “reactive shuttles.” Direct, like-for-like quantitative comparisons of “reactivity efficiencies” between biofouled MNPs and natural particulates remain limited because studies rarely normalize to surface area, biofilm biomass, and particle abundance. Available evidence also indicates that natural particles can dominate in some cases; for example, river sediment was more effective than PS and LDPE pellets for the adsorption of arsenic species from water. This supports our revised framing that MNPs are “distinct” primarily in their mobility/persistence and exposure routing, rather than universally more reactive than sediments.^13^ However, we currently lack frameworks to quantify whether plastics represent a negligible pathway or a disproportionately important one in global contaminant cycling.^10,41^

Speciation-focused monitoring is only interpretable if sampling and extraction preserve the environmentally experienced entity: polymer surface + eco-corona + plastisphere matrix + associated metal pools. Many commonly used extraction workflows (strong oxidants/alkali, aggressive digestion, high-shear processing, and prolonged drying) risk removing EPS/corona layers and perturbing redox-sensitive species, thereby converting a biofouled particle into a different analytical entity. I, therefore, highlight “native” isolation as a priority: gentle size/density separation with rigorous blanks, minimal chemical digestion when interfacial chemistry is the target, low temperature/dark handling, and complementary characterization of eco-corona/plastisphere composition. Soft separation approaches such as asymmetric flow field-flow fractionation (AF4) coupled to multidetectors have been demonstrated for eco-corona interrogation and can be extended to more representative polymers (e.g., PET-based nanoplastics) rather than relying only on pristine PS beads.^42,43^

It also remains unclear under which environmental contexts biofouling reactivity^44,45^ —in sediments, surface waters, polar regions, or tropical estuaries, and how transformations vary across particle size classes from nanoplastics to millimeter fragments. For example, MNPs may host distinct microbial consortia and biomolecules and present larger reactive surface areas, but their role in contaminant speciation remains virtually unexplored. Equally absent are toxicological validations linking transformation products to organism-level outcomes. For instance, whether As (V) generated by plastisphere-derived ROS is indeed more harmful to phytoplankton or whether Pb–EPS complexes enhance trophic transfer in mussels or fish has yet to be systematically tested. Without bridging these mechanistic insights into organismal and ecological effects, the true significance of plastisphere-driven transformations for ecosystems and human health remains speculative.

Further, the **temporal dynamics** of these processes is poorly understood. How diurnal light cycles, storm-driven resuspension, seasonal changes in microbial communities, or climate-driven warming modulate transformation rates has not been examined, yet such factors could be decisive in determining hotspots and fluxes of reactivity. In an era of accelerating environmental change, integrating these temporal and climate-linked dynamics into plastisphere research is crucial.^8^ These gaps highlights that the role of MNPs in contaminant cycling remains unresolved; they may be minor contributors compared to natural matrices, or their persistence and mobility may confer disproportionate influence in certain environments. Closing these knowledge gaps will require interdisciplinary approaches that couple speciation analytics, ecotoxicological testing, field surveys across diverse contexts, and predictive models that explicitly account for plastic-associated reactivity. These unresolved questions directly shape what can be monitored, compared, and regulated with confidence (Figure 1).

Priority validation tests include (1) incubating plastic coupons/MNPs to develop plastisphere biofilms, then adding stable-isotope tracers to quantify transformation rates (not only sorption); (2) mapping microenvironment gradients (pH/Eh/O_2_) across the plastisphere using microelectrodes; (3) measuring oxidation state/speciation directly on biofouled MNPs (e.g., X-ray absorption spectroscopy (XAS)­including X-ray Absorption Near Edge Structure (XANES) and linking outcomes to EPS chemistry; and (4) coupling these measurements to functional markers (e.g., genes associated with Hg methylation potential) to connect community function to observed speciation changes.

To avoid overattribution of effects to MNPs, future studies should include **natural-particle baselines** (sand/clays/sediments) and report outcomes normalized to surface area and biofilm biomass, alongside system-level mass balances. Recent work comparing biofilm-covered HDPE MNP and biofilm-covered sand found **similar transfer efficiency** of sorbed PCB-153 to a benthic grazer, illustrating why baseline comparisons are essential for judging when MNPs “outweigh” natural particles.^12^

Another critical barrier to decision-grade evidence is the shortage of representative, traceable, and well-characterized MNP test/reference materials, including weathered and biofouled states. Without such materials, laboratories remain locked into noncomparable simulated systems and cannot robustly validate extraction, speciation, and toxicology workflows.^46,47^ I, therefore, call for coordinated development of benchmark MNP materials and interlaboratory exercises that quantify recovery, artifacts, and uncertainty across methods.

Further, a key next step is direct side-by-side testing of biofouled MNPs against natural particulates (e.g., sediments, clays, organic detritus, and natural biofilms) under matched hydrodynamic and chemical conditions. This is essential to determine when particle-associated transformations on plastics are negligible relative to background particle processes and when plastics become disproportionately important because of mobility, persistence, or biological uptake pathways.

## Call to Action

Current environmental policies and water-quality regulations generally focus on reducing plastic litter counts or limiting total heavy metal concentrations, but they do not account for the in situ transformations induced by the plastisphere. For example, biofouled MNPs in sediment may methylate mercury or oxidize arsenic, creating more toxic species without any new emissions of those metals. This gap between science and policy means that we could be underestimating the true risk of plastic pollution.

International efforts offer a chance to bridge this gap. The United Nations is negotiating a Global Plastics Treaty to end plastic pollution.^41,48^ To be effective, such agreements should explicitly address MNPs-associated contaminants and their chemical transformations. Scientists have stressed that existing national laws are insufficient and that the treaty is a “tangible opportunity” to coordinate action globally. It should commit not only to reducing plastic debris but also to monitoring and mitigating microplastic–pollutant interactions. Notably, a recent expert review urged that certain plastics (e.g., MNPs laden with hazardous chemicals) be classified as persistent, bioaccumulative and toxic (PBT) substances.^1^ Adopting such designations in policy would compel more precautionary handling of biofouled plastics as hazardous waste rather than benign debris.

Precautionary measures can be integrated into existing frameworks. For instance, water and sediment quality guidelines could be updated to consider speciation of pollutants on MNPs surfaces (not just total concentrations). Environmental monitoring programs should employ advanced speciation analysis (XANES, High-Performance Liquid Chromatography coupled with Inductively Coupled Plasma Mass Spectrometry (HPLC-ICP-MS), etc.) on recovered plastics, as recommended in our Call to Action, to detect any formation of more toxic metal species. Remediation strategies might prioritize hotspots like estuaries or wastewater outfalls, where dense microplastic biofilms could be “reactivating” legacy metals. While advanced speciation tools (e.g., XAS, HPLC–ICP–MS) provide decision-critical detail, they are costly and not yet feasible for routine deployment across all monitoring networks. I, therefore, propose a staged approach: **near-term** screening (polymer ID, particle abundance, and total metals) with **targeted speciation** at priority hotspots and sentinel biota, alongside **longer-term** investment in reference laboratories, standard protocols, and interlaboratory comparability to enable scalable speciation monitoring.

Equity and accountability are also critical dimensions. UNEP’s report *Neglected: Environmental Justice Impacts of Marine Litter and Plastic Pollution* (2021)^49^ highlights that disadvantaged communities, particularly in the Global South and near waste or recycling sites, face disproportionate exposure to plastic-linked contaminants. A *Nature* editorial (2023)^50^ similarly emphasized that countries must be held accountable for reducing plastic leakage, which now exceeds 400 million tonnes annually. Embedding such accountability and justice considerations into the Global Plastics Treaty would ensure that interventions do not merely reduce global totals but also alleviate burdens on the communities most at risk.

Global agencies are beginning to recognize these issues. The OECD’s *Global Plastics Outlook* ^51^ warns that plastic leakage (including microplastics) will double by 2060 without ambitious policies, potentially exacerbating the distribution of particle-bound pollutants. Likewise, the Food and Agriculture Organization (FAO) has highlighted that MNPs in seafood can sorb and transfer toxins into the food chain underscoring food safety implications.^52^ These insights should inform not only pollution control and waste management policies, but also chemicals regulation (e.g., adding plastic-associated chemical risks into chemical safety assessments). Together, these policy developments provide an entry point for incorporating plastisphere-driven transformations into monitoring and regulation.

## Implications

A key uncertainty is the scale of their impact. Are these plastic-associated reactions only minor pathways compared to natural sediment or water-column processes, or could they significantly alter local and even global contaminant cycles? Given that MNPs have permeated essentially every corner of the planet and entered over 1,300 species (including our own bodies),^41^ even a small enhancement in contaminant mobility or toxicity could have widespread ramifications. At the global scale, natural particulates (sediments, clays, and detritus) likely dominate the total reactive surface area and many baseline transformation processes; therefore, biofouled MNP effects are expected to be heterogeneous rather than universally dominant. However, global-scale significance can still arise if MNPs consistently create local hotspots that amplify mobility or toxicity at points of exposure (e.g., ingestion, benthic contact, and food-web transfer), especially given the ubiquity of MNPs across ecosystems and biota.

To confront this challenge, we identify three priorities: (1) Speciation-focused monitoring that resolves forms, not just totals, using tools such as XAS and MS for metal–ligand complexes; (2) mechanistic validation in controlled and mesocosm studies using aged, biofilm-covered plastics under realistic sunlight, temperature, and redox variability to test whether MNPs amplify or simply mirror natural-particle processes; and (3) risk integration, embedding plastisphere reactivity into cycling and risk models where evidence supports it (e.g., adding MNP terms to Hg cycling in wetlands). Policymakers should recognize that plastic pollution is also a chemical-transformation issue, not only a litter issue.^1^ Addressing plastisphere transformations will require coordinated input from chemists, microbiologists, toxicologists, ecologists, food safety and health scientists, climate scientists, social scientists, and policy experts to connect mechanisms with ecosystem effects, exposure pathways, and governance. As highlighted in Yates et al. (2025), only cross-sectoral integration can match the systemic complexity of plastic pollution.^53^

In conclusion, as we enter the “Plasticene” era of pervasive plastic pollution, we must broaden our perspective on what these materials do in the environment.^54^ The coming years will be crucial for determining to what extent biofouled MNPs enhance or alter the fate of contaminants relative to natural processes. By proactively investigating and accounting for these **overlooked transformations**, we can better protect ecosystems and public health.

## Data Availability

No original data was generated for this perspective article.
